# A Community-Engaged Approach for Assessment of Cortisol Dynamic Range and Multilevel Chronic Stress in African Americans: FAITH! Heart Health+ Ancillary Study

**DOI:** 10.2196/69604

**Published:** 2025-12-01

**Authors:** Robin Ortiz, Joshua Joseph, Matthew P Johnson, Lainey Moen, Mathias Lalika, Clarence Jones, Irina Bancos, Lisa A Cooper, Sharonne N Hayes, Christi A Patten, LaPrincess C Brewer

**Affiliations:** 1 Departments of Pediatrics and Population Health New York University Grossman School of Medicine New York, NY United States; 2 Institute for Excellence in Health Equity New York University Langone Health New York, NY United States; 3 Division of Endocrinology, Diabetes and Metabolism The Ohio State University Columbus, OH United States; 4 Department of Quantitative Health Sciences Mayo Clinic Rochester, MN United States; 5 Department of Cardiovascular Medicine Mayo Clinic Rochester, MN United States; 6 Hue-Man Partnership Minneapolis, MN United States; 7 Division of Endocrinology, Diabetes, Metabolism and Nutrition Mayo Clinic Rochester, MN United States; 8 Department of Medicine Johns Hopkins University Baltimore, MD United States; 9 Department of Psychiatry and Psychology Mayo Clinic Rochester, MN United States

**Keywords:** cortisol, stress, racism, community engagement, African Americans

## Abstract

**Background:**

Chronic stress in African American individuals is multilayered amid the context of experiences of racism and discrimination. Cortisol dynamic range (CDR) may be an indicator of chronic stress, but CDR is understudied in African American populations compared with White populations, and is hypothesized to differ by sex.

**Objective:**

Using a community-engaged approach within the Fostering African-American Improvement in Total Health! (FAITH!) Heart Health+ ancillary study, we assessed the feasibility of participant-centric CDR collection, and its association with measures for individual, interpersonal, and structural stress and exposure to racism in medically underserved African American women and men.

**Methods:**

Participants residing in the Minneapolis-St Paul and Rochester, Minnesota areas provided survey data (everyday discrimination, perceived stress, mood, sleep quality, and high effort coping measures), and saliva samples (morning and afternoon) via at-home, self-collection kits for cortisol measurement. CDR was calculated as a difference in log cortisol levels (ie, log of the cortisol diurnal peak-to-nadir ratio). Geospatial Area Deprivation Index and the distance lived from George Floyd Square in Minneapolis were calculated. Linear regression examined the association between CDR and outcome variables.

**Results:**

Of consented participants (n=53), 70% (37/53) provided cortisol samples. The final analytic sample included 32 participants with complete and physiological diurnal cortisol curves (mean age 57.5 years, 62.5% [20/32] women). Lower (less dynamic) CDR in women (n=20) was associated with greater perceived stress (β=–0.07, *P*=.01), greater anxiety (β=–0.06, *P*=.01), higher Superwoman Schema score (β=–0.02, *P*=.04), and greater distance from George Floyd Square (β=–0.02, *P*=.01). No associations were observed in men (*P*>.05).

**Conclusions:**

The current results suggest that CDR from participant-led saliva collection is feasible and may serve as a biomarker of chronic and physiological stress in African American women, particularly those residing in underresourced areas.

## Introduction

Racism may be experienced at different points along the spectrum between an individual and their environment. In addition to interpersonal experiences of racism, systemic or structural racism can impact individual and collective experience. Structural racism can be defined as, “processes of racism that are embedded in laws (local, state, and federal), policies, and practices of society and its institutions that provide advantages to racial groups deemed as superior, while differentially oppressing, disadvantaging, or otherwise neglecting racial groups viewed as inferior” [1,2]. Historical examples that impact current manifestations of structural racism include redlining, school segregation, and inequitable practices in medical care and research. For individuals in marginalized populations, each layer of exposure to racism may contribute to physiologic dysregulation and, ultimately, health disparities. Physiologic dysregulation as a result of chronic exposure to harmful stressors, which may originate from different sources contextualizing one’s lived experience and environment (eg, psychosocial, infectious, chemical, and environmental), may be referred to as “toxic stress” [3]. In a conceptual framework of toxic stress and the impact of racism, exposures that impact health may be present at the individual and interpersonal level, through individual everyday experiences of stress and racism, or the structural level, such as through the built environment [4]. Therefore, community-engaged research studies that aim to holistically investigate the environmental and physiological pathways for the impacts of racism on health must be inclusive of multimodal methodology [5]. For example, study designs may aim to consider self-reported metrics, questionnaires, and self-collected biospecimen data alongside contextual neighborhood factors and measures of the structural environment [5]. Cortisol may play a role in the physiology underpinning toxic stress, as it is the effector stress hormone of the physiological stress pathway (eg, the hypothalamic-pituitary-adrenal [HPA] axis), which has a natural diurnal rhythm. Cortisol dynamic range (CDR) is a specific measurement that captures diurnal cortisol measured as a difference between the peak (highest point) and trough (lowest point) of cortisol concentration over the course of the day [5,6]. Recently, it has been demonstrated that CDR may be a feasible and promising measure in community-engaged research to assess toxic physiological stress burden [5]. For example, experiential and economic adversities in childhood have been associated with lower cortisol range [6]. Moreover, adverse health outcomes have also been associated with CDR. For instance, a lower range in diurnal cortisol (flatter CDR) has been associated with lower cognitive functioning and behavioral manifestations of cognitive impairment, which may indicate that lower CDR could be a precursor of cognitive decline in the population [7,8]. Including assessments of cortisol (biology), coping, and experiences by self-report questionnaires and social context (geocoded variables), our study uses a biopsychosocial approach to understanding the impact of minority stress on health [9].

The manifestation of toxic stress physiology (eg, the toxic stress response) depends on the presence of buffering factors and resources, including supportive relationships and environments [4]. One aspect of the buffering of internalized racism may occur through individual emotional regulation and coping. However, sex differences may exist for such factors, specifically for the impacts of racial discrimination experienced by African Americans [10]. For example, the Superwoman Schema (SWS) conceptual framework identifies that African American women may respond in the face of challenge with a great manifestation of strength, resistance, determination, and obligation to others, even when faced with limited resources [10-12]. African American men may build a response to exposure to racism with a heightened sense of vigilance and effortful coping, or John Henryism [10,13,14]. Importantly, the literature also supports sex-specific cortisol-related physiological responses to stress, with women having greater overall cortisol levels after stress and potentially more dynamic cortisol responsiveness [10,15,16]. There have been studies that have assessed sex-based differences in other cortisol measures, such as cortisol awakening response and morning cortisol, demonstrating a higher rise or levels, respectively, in females compared with males [17-19]. Other studies have emphasized the unique role of chronic stress in association with dysfunctional HPA axis response, specifically, flattening of the diurnal cortisol curve in African American women [20,21]. Further, this cumulative stress in African American women of child-rearing age has potential physiological impacts on HPA function in future generations [22].

Diurnal cortisol, while important to study, may be especially susceptible to variation in the methodology of collection, which is a particular challenge in community-engaged work [5]. However, a recent feasibility study of the collection of cortisol as part of a larger neighborhood environment study in a predominantly African American, low-income urban cohort demonstrated that the use of CDR, may be less vulnerable to temporal variation over the course of the day [5]. This is owing to the fact that CDR requires only measures of cortisol concentrations at 2 points (one each in the highest and lowest range) in the day and is less dependent on specific times of measurement, as other cortisol measures are, such as the awakening cortisol response that must be calculated over the first 30-45 minutes of the day [5]. While CDR measurement is based on peak and trough, cortisol does decline over the course of the day into the evening. However, prior methods have demonstrated consistency of measurement and utility of CDR for collection in the earliest part of the day (period of rise) and later part of the day (period of decline) [5,6]. However, this study relied on resource-intensive, community-based data collection of saliva samples by study team members within the homes of participants. This led to the conclusion that further refinement and standardization of methodology, especially for participant-led self-collection of biospecimen data, in multimodal community-engaged studies, which require community member trust in research and the study team, is necessary [5]. Further, while this study speculated about the potential for CDR to serve as a biological indicator of the stress of racism and discrimination experienced by individuals in marginalized communities, it did not directly measure experiences of racism or discrimination in participants.

This study aimed to (1) explore the feasibility of measurement of CDR using salivary cortisol samples collected at home and (2) investigate the association of CDR with measures of multilevel chronic stress exposures (individual, interpersonal, and structural racism) and coping. To do this, it harnessed existing data collected within the context of a community-based clinical trial of medically underserved African Americans informed by the National Institute on Minority Health and Health Disparities (NIMHD) Research Framework, which integrates multi-dimensional aspects of influences on health outcomes [23]. The baseline data of this clinical trial were applied for the current ancillary study after the collection of self-reported individual measures of stress, interpersonal exposure to, and geospatial data representing exposure to structural racism. The study cohort was located in Minneapolis-St Paul and Rochester, Minnesota, and surrounding areas. Therefore, a pertinent and unique exploratory proxy for systemic racism was included as proximity to George Floyd Square, the site of the 2020 police killing of Mr George Floyd. Importantly, the social unrest and psychological impacts in the population persisted well after the murder of George Floyd [24], and the collection of data relevant to the parent clinical trial for this ancillary study was ongoing during the court trials of Minneapolis law enforcement.

To support the refinement of community-engaged approaches to biospecimen collection in multimodal research methodology, this study also aimed to build on a prior feasibility study related to the applications of CDR [5]. Specifically, we assessed the feasibility of participant-led self-collection with self-dependent return of the biospecimens by mail (prior study relied on study team members in the field) for CDR measurement using just 2 timepoints of saliva sample collection (prior study collected at 4 timepoints). Our central hypothesis was that methodology for assessment of CDR, specifically, would be feasible as evidenced by (1) a high percentage of participant engagement in sample collection for just 2 samples needed for measurement calculation (>50%), and (2) a majority of CDR values showing directional consistency with physiologically expected pattern of diurnal cortisol (eg, morning cortisol levels as greater than late day cortisol levels). Demonstration of the ability to consider the use of CDR as a measure of the multifactorial impacts of racism, including individual, interpersonal, and structural levels, on physiology will enable its use as a measure in future community-based studies following the multimodal framework of the NIMHD that outlines corresponding levels and domains of influence on health outcomes [23]. Further, demonstration of the feasibility of the methodology of CDR measurement used in this study could support an approach to include the collection of such a biomarker amid aims for the decentralization of clinical trials since the COVID-19 pandemic [25,26].

After assessing feasibility, the study aimed to conduct an exploratory, hypothesis-generating analysis informed by the literature. Prior literature has demonstrated that cortisol responsiveness in African American women may be more dynamic in the face of stress [10,27]. Our own work recently suggested an association between SWS and perceived stress among African American women of high cardiometabolic risk within the FAITH! (Fostering African-American Improvement in Total Health!) Heart Health+ (HH+) Study [28]. Therefore, it was further hypothesized that greater stress, exposure to racism at the interpersonal and structural levels, and poorer sleep quality would be associated with lower CDR (less dynamic or adaptable cortisol or stress response) in African American women [28]. Further informed by the literature, we hypothesized that the greater manifestation of the SWS in African American women specifically would be inversely associated with CDR (eg, associated with lower or flatter CDR). Lastly, exploratory analyses were completed in African American men of the association between stress exposure variables and CDR, adding a hypothesis that John Henryism-type high effort coping would be associated with lower CDR, specifically in African American men.

## Methods

### Ethical Considerations

The study received approval from the Mayo Clinic Institutional Review Board (IRB No. 21-011103). All study procedures were conducted in accordance with the ethical standards of the institutional and national research committees and with the principles outlined in the Declaration of Helsinki. All participants provided electronic written informed consent before participating in the study. Participants received US $50 for completing the electronic survey, US $50 for health assessment laboratory studies, and biospecimen transport expenses were covered.

### Study Design, Community-Based Participatory Approach, and Theoretical Framework

This cross-sectional analysis was conducted as part of a decade-long community-based participatory research partnership (CBPR). This cross-sectional analysis was conducted as part of a decade-long CBPR through the FAITH! Cardiovascular Health and Wellness Program. In response to the horrific tragedy of the killing of George Floyd by law enforcement in 2020, FAITH! academic and community partners conceptualized the FAITH! HH+ ancillary study in 2021 to examine the multifactorial effects of the COVID-19 pandemic and adverse psychosocial and SDOH factors stemming from heightened racial tensions on the cardiovascular health of African Americans. The FAITH! Program Community Steering Committee, consisting of diverse community leaders from the Minneapolis-St Paul and Rochester, Minnesota, areas, collaborated with the study team in compiling relevant psychosocial factors and measures for assessment in this study (via 2 web-based group meetings and follow-up weekly study team meetings from Summer 2021 to Winter 2022) [29]. The Community Steering Committee reviewed all proposed validated survey instruments and selected those deemed most appropriate and meaningful to the study population. The Community Steering Committee also assisted in the development of all recruitment materials and strategies to ensure their cultural appropriateness (eg, representation of African Americans in visuals, lay-friendly language, and multimodality formatting [social media, email, and flyers]; Figure S1 in Multimedia Appendix 1). Study participants were recruited to participate in the HH+ study as an extension of an overarching randomized control trial of a behavioral lifestyle intervention to support cardiovascular health in African Americans (FAITH! Trial, N=83 [ClinicalTrials.gov unique identifier NCT03777709]). The HH+ study integrated a community-engaged approach for the collection of psychosocial and environmental measures related to cardiovascular health. Details of the FAITH! Trial and HH+ study designs and recruitment processes have been published [30,31].

The NIMHD Research Framework was applied for the refinement of the community-engaged approach and to inform the multidimensional aspects of this study [23]. This framework integrates individual, interpersonal, community, and societal levels of influence through the domains of biological, behavioral, physical/built environment, sociocultural environment, and health care system to inform health outcomes. These levels and domains of influence informed the design of data to inform the measured exposure variables described below (see Exposures subsection). The inclusion of CDR, specifically, draws on the biological domain of this framework and is informed by the biopsychosocial approach to understanding the impact of minority stress on health [9].

### Data Collection

Each participant completed an electronic questionnaire to self-report their sociodemographics and psychosocial stress, and self-collected salivary cortisol samples with home kits.

### Salivary Cortisol

With community input, the study team collaborated with Mayo Clinic Laboratories (Rochester, Minnesota) staff and endocrinology specialists (IB and JJ) to devise a participant-centric protocol for self-collection of salivary cortisol. This modality was mutually selected among the academic-community partner team as a potentially more convenient means for participants to provide cortisol for CDR assessment (vs venipuncture or hair). Participants were asked to collect saliva samples using the Sarstedt Salivette kit (Nümbrecht, Germany) and received detailed instructions on proper technique following Mayo Clinic protocols [32]. Specifically, participants collected samples at 2 different time points over the course of a day, including the morning (collection 1: 7-9 AM) and evening (collection 2: 3-5 PM). Instruction materials emphasized recording the exact time of sample collection on the collection tube. Participants were asked to place samples at room temperature in a supplied biohazard bag and to return samples by mail in a prepaid shipping envelope to the Mayo Clinic Laboratories (Rochester, Minnesota), where samples were processed for cortisol concentration [32]. Participants also had the option of returning their samples in-person at centralized community-based venues to a mobile clinical research unit team of trained nursing staff. As cortisol collection for CDR measurement was part of a feasibility study in a larger overarching RCT, there were no exclusion criteria for factors that may impact cortisol levels, such as night shift working or taking steroid or beta-blocker medications.

### Outcome

#### Cortisol Dynamic Response: Toxic Stress Response

The toxic stress response, the physiological burden of chronic stress, was assessed by CDR and calculated as in prior literature [5,6,8]. The peak (morning) and nadir (evening) [5], cortisol levels were log transformed, and the difference was calculated. The difference in log-cortisol measures translates into the log of the cortisol diurnal peak-to-nadir ratio. A higher value of CDR indicates a more dynamic diurnal response, which has been shown to correlate with lower self-reported stress and better health outcomes, while a lower value of CDR indicates a more suppressed or flattened diurnal response [5,6,8]. Prior studies have found the average CDR to range from ≈1.7 to 1.9 log-nmol/L [5,6,8], where, for reference, 1.85 log-nmol/L equates to an approximate 6 to 1 peak-to-nadir ratio [6].

#### Exposures

**Figure 1:**
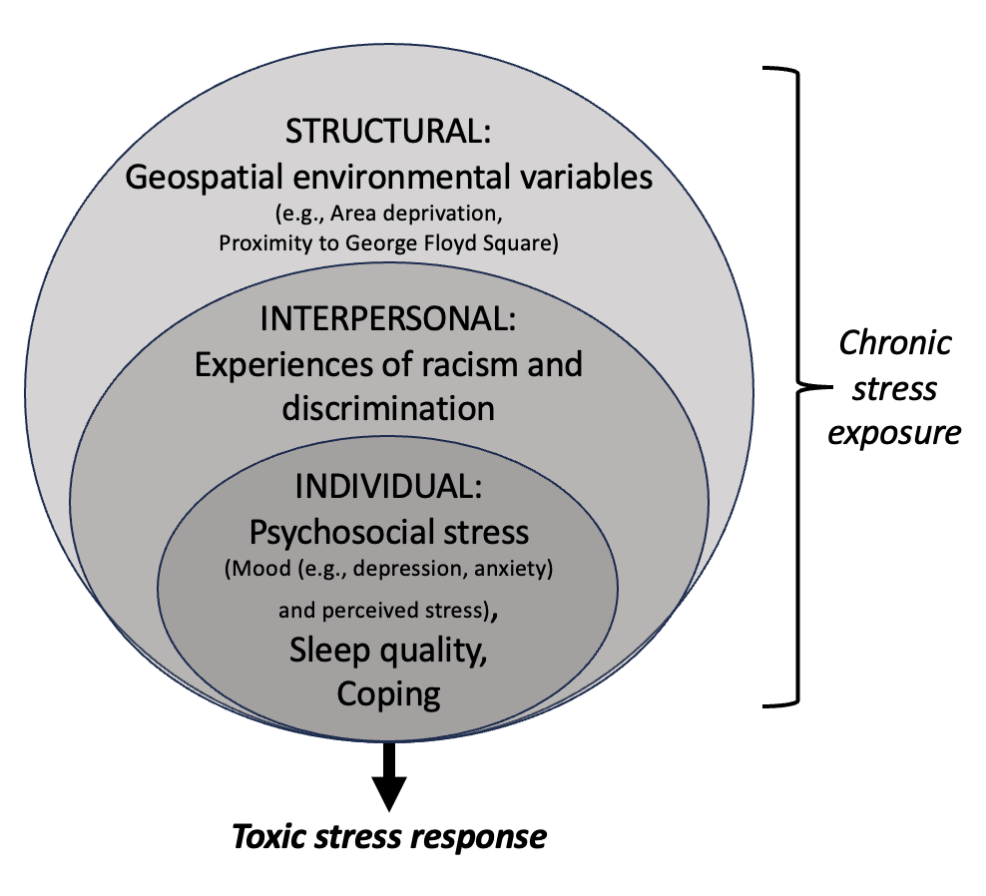
Study conceptual framework. This study is designed to assess the association between multilevel sources of chronic stress and the toxic stress response measured through Cortisol dynamic range (CDR). Multilevel chronic stress exposure measured in this study included those at the individual, interpersonal, and structural levels.

Given the conceptual framework of the multilevel impact of toxic stress and racism informed by the NIMHD Research Framework [23], exposure data were collected at the individual, interpersonal, and structural levels (Figure 1).

#### Psychosocial Stress

Psychosocial stress was measured by the Global Perceived Stress Scale (GPSS) and scales for depression and anxiety symptoms (Center for Epidemiological Studies Depression [CES-D] and Patient-Reported Outcomes Measurement Information System [PROMIS] short form) [33,34]. The GPSS has been used in studies of African Americans and assesses multiple domains of stress experiences over the last month, including those related to employment, legal concerns, and discrimination [35-37].

#### Sleep Quality

Sleep quality was measured by the Pittsburgh Sleep Quality Index, which offers a global sleep quality score ranging from 0 (better) to 21 (worse) [38].

#### High-Effort Coping

Coping in African American women as described by the SWS conceptual framework [11,12] has been conceptualized in a 35-item questionnaire (Giscombe SWS Questionnaire), which includes 5 domains: obligation to present an image of strength, obligation to suppress emotions, obligation to help others, resistance to vulnerability, and intense motivation to succeed even in the face of limitations [39]. Each SWS domain was assessed on a scale of 0 (not true) to 3 (true all the time). A total SWS score was calculated by summing across all 35 items, and a mean overall score was calculated across all participants, where a higher score correlates to greater endorsement of SWS characteristics (range 0 [lowest coping] to 105 [highest coping]) [39].

John Henryism was measured in both African American women and men in the HH+ Study, but it was used for analysis in this study, intentionally stratified by sex based on literature indicating these measures may be particularly relevant to the manifestations of high effort coping observed in African American men [10,14]. The John Henryism scale is a sum with a greater score indicating greater active effort in coping (range 12-60) [40].

#### Experiences of Racism and Discrimination

Interpersonal everyday experiences of discrimination were measured with the Everyday Discrimination Scale, with a total score reported as a mean (range 0-36) [41,42]. While averaging item scores into a mean may mask variation in the type or frequency of discriminatory experiences, the use of a mean composite score is consistent with the analytic approach used in the majority of studies using this instrument. Notably, multiple validated studies among African American populations have demonstrated a consistent association between the mean Everyday Discrimination Scale score and a range of adverse health outcomes [43-47]. Accordingly, we adopted this approach to align with established methodology and to facilitate comparability with prior research.

#### Geospatial Environment Variables

A general geospatial variable included in the study consisted of the Area Deprivation Index (ADI), based on a census block of 17 indicators of poverty [48,49]. A greater ADI indicates greater neighborhood deprivation. Proximity to social unrest, calculated as the distance in kilometers between participants’ residential home addresses and George Floyd Square in Minneapolis, Minnesota, was used as an exploratory proxy for exposure to structural racism.

### Statistical Analysis

#### Feasibility of Measurement of CDR

We first assessed the proportion of participants completing saliva collection as instructed, and defined a high engagement as above 50%, based on literature demonstrating that in population-wide studies including saliva collection, the rate of biospecimen return ranged from 15% to 80% (averaging approximately 42%) [50]. We also assessed the quality of biospecimen return by calculating the number of participants who provided saliva within increments of 30 minutes from the recommended collection times, and the number of participants who had a negative value for the difference in concentration of salivary cortisol (inverse) over the 2 collection timepoints (collection 2 to collection 1). As it is not physiologically feasible for one’s cortisol curve to be higher in the evening than in the morning, a negative value would indicate improper collection (eg, mislabeling of the time of collection of the samples), or physiologically reversed diurnal rhythms (eg, nightshift work, or regular pattern of daytime sleep rather than nighttime). To understand potential challenges to biospecimen collection, characteristic comparisons were made for the sample by those who did versus did not complete biospecimen collection, and by those who had a physiologically expected CDR with those who had inverse CDR measures. Individuals with an inverse CDR were excluded from the analyses testing associations between psychosocial measures and CDR.

#### Exploratory Analysis: Stress, Racism, Coping, and Cortisol Dynamic Range

Exploratory linear regression analyses were conducted to test the hypothesis that exposure to chronic stress at different levels (individual, interpersonal, and structural), as per Figure 1 (independent variables each tested in separate regression models), would be associated with flatter CDR (outcome, dependent variable for all regression models). Given the hypothesis that associations between exposures to stress would impact CDR differently for men and women, and to test the hypothesis that sex-specific coping mechanisms (SWS for women vs John Henryism for men) would be associated with CDR, analyses were stratified by sex. Determination of potential inclusion of covariates was made based on descriptive analyses (*t* test) comparing mean values for sociodemographic variables by sex (female vs male) and by CDR dichotomized at the median (high vs low CDR). Graphical depiction demonstrated the comparison of the range of high effort coping scores by high versus low CDR. Cronbach α was calculated within each individual and interpersonal stress exposure variable to assess the internal reliability within our sample. All analyses were considered statistically significant at a *P* value of <.05 with no adjustments for multiple comparisons given the exploratory, hypothesis-generating nature of these analyses.

As distance from George Floyd Square is a unique variable in this dataset, a variable-specific exploratory analysis to lend to the interpretability of this variable as a potential measure of structural racism was conducted. We tested the hypothesis that closer proximity to George Floyd Square would be associated with lower socioeconomic status (lower income) in this study population. This was done by comparing the dichotomous (at the median) variables of income by distance using chi-square. Additionally, the difference between stress levels and coping (GPSS and SWS for African American women) was explored by distance using independent samples *t* tests. For these exploratory interpretation analyses, observations considered significant for the purposes of interpretation for *P* values <.10. Given the hypothesis that distance from George Floyd Square may be a proxy for racism through inequitable distribution of resources, a sensitivity analysis (of the regression model applying it as an independent variable with CDR as the dependent variable), was conducted including adjustment for income.

All statistical analyses were conducted using Stata SE (version 18.0; StataCorp, 2023 statistical software).

## Results

### Participants’ Characteristics and Feasibility of CDR Measurement

**Figure 2:**
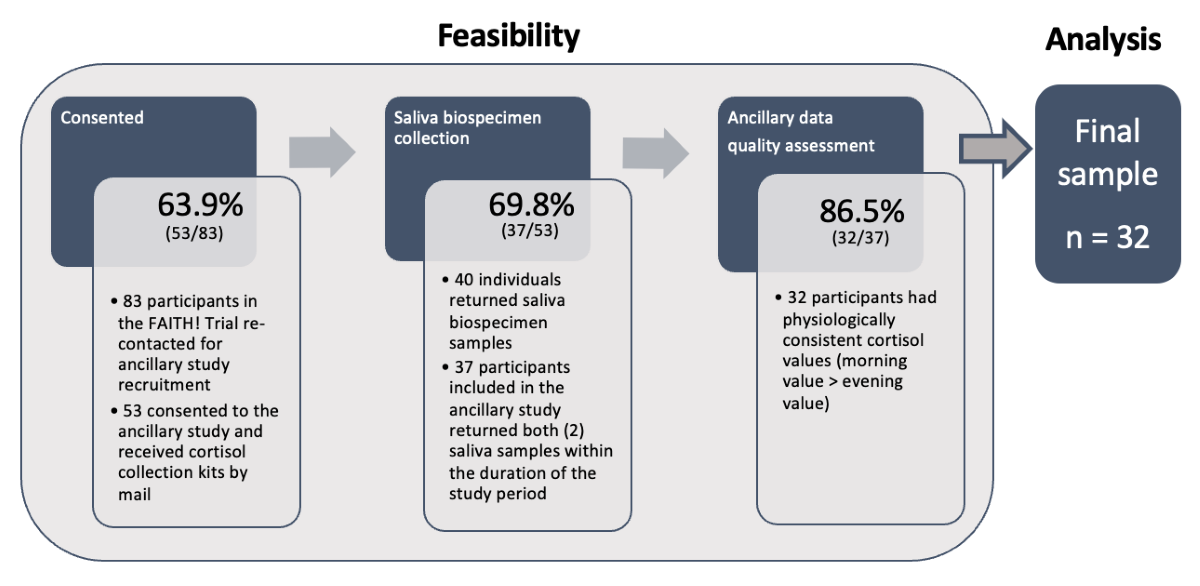
Study flow diagram. The ancillary FAITH! Heart Health+ (HH+) Study was part of an overarching randomized controlled trial of an intervention to support cardiovascular health in African Americans. A number of individuals in the FAITH! HH+ Study were recontacted for enrollment in the ancillary study (n=83). Of these, 53 consented and were mailed saliva collection kits, and 32 had complete and physiologically consistent cortisol data (cortisol at 2 timepoints with morning cortisol value greater than evening cortisol value). This cohort of individuals (n=32) was included in analyses between exposure to psychosocial stress, coping, and racism and cortisol dynamic range. FAITH!: Fostering African-American Improvement in Total Health!.

The final analytic sample included 32 participants, all with 2 samples available for analysis and physiologically appropriate cortisol values (morning > evening). Of the FAITH! Trial cohort participants (N=83) recontacted for participation in the HH+ study, 53 (63.9%) were consented and received cortisol collection kits by mail (Figure 2). Another 40 (75.5%) returned samples (29 in person, 11 by mail), and 13 were not returned; 37 participants returned both (2) saliva samples within the duration of the study period (69.8% of those consented).

Of the 37 participants returning both (2) saliva samples, 33 had the detailed time of collection data available. Most provided a morning cortisol sample within 1 hour of the recommended time of 8:00 AM (between 7 and 9 AM; 29/33, 87.8%), with 27.3% (9/33) providing a sample within 30 minutes (between 7:30 and 8:30 AM). For the afternoon sample, all participants provided a sample at or after 3 PM (16/33, 48.5% by 3:30 PM and 22/33, 66.7% by 4 PM).

The majority of participants had samples that aligned with physiological diurnal cortisol curves, with a lower cortisol value at collection 2 compared with collection 1 (32/37, 86.5%); however, 5 participants had higher cortisol at collection 2. The 32 participants with physiologically consistent CDR were included in analyses for associations between exposures and CDR.

Participant sociodemographic characteristics are displayed in Table 1. The mean age of this sample was 57.5 (SD 11.6) years. The majority were women (20/32, 62.5%). While the majority reported an annual household income >US $75,000, there was a relatively even split among the lower gradations of income, with 5 that did not disclose (Table 1). Most participants were college graduates or higher. Internal consistency for the individual and interpersonal stress exposure variables ranged from 0.67 (0.49-0.81) to 0.93 (0.90-0.96).

**Table 1:** Sociodemographic characteristics, chronic stress measures, and cortisol dynamic range of Fostering African-American Improvement in Total Health! (FAITH!) Heart Health+ study participants.

				Variables
**Demographic characteristics (N=32)**				
	**Sex (n=32), n (%)**			
		Men		12 (37.5)
		Women		20 (62.5)
	**Age (years; n=31)**			
		Mean (SD)		57.5 (11.6)
		Range		30-86
	**BMI (kg/m^2^; n=31)**			
		Mean (SD)		36.2 (8.1)
		Range		22.7-56.4
	**Annual household income (US $; n=31), n (%)**			
		<50,000		12 (38.7)
		≥50,000		14 (45.2)
		Not disclosed		5 (16.1)
	**Education (n=30), n (%)**			
		≤High school		3 (10.0)
		>High school		27 (90.0)
**Stress exposure variables**				
	**Individual**			
		**Psychosocial stress (GPSS^a^; n=30)**		
			Mean (SD)	6.5 (5.1)
			Range	0-24
		**Anxiety symptoms (n=29)**		
			Mean (SD)	11.0 (5.5)
			Range	8-31
		**Depressive symptoms (n=31)**		
			Mean (SD)	15.3 (4.4)
			Range	9-25
		**Sleep quality (PSQI^b^; n=25)**		
			Mean (SD)	6.4 (4.44)
			Range	1-16
		**High effort coping: Superwoman Schema, women only (n=20)**		
			Mean (SD)	55.5 (17.4)
			Range	20-80
		**High effort coping: John Henryism (n=31)**		
			Mean (SD)	48.0 (6.09)
			Range	35-58
	**Interpersonal**			
		**Everyday discrimination (n=32)**		
			Mean (SD)	21.8 (7.5)
			Range	9-40
	**Structural**			
		**Area deprivation (ADI^c^; n=31)**		
			Mean (SD)	5.0 (2.47)
			Range	1-10
		**Distance from George Floyd Square (km; n=31)**		
			Mean (SD)	23.5 (34.5)
			Range	1.5-115.4
		**Cortisol dynamic range (n=32)**		
			Mean (SD)	1.39 (0.7)
			Range	0.02-2.8

^a^GPSS: Global Perceived Stress Scale.

^b^PSQI: Pittsburgh Sleep Quality Index.

^c^ADI: Area Deprivation Index.

Consented individuals who did not complete biospecimen return were younger (48.9, SD 12.1 vs 57.5, SD 10.9 years, *P*=.009) and reported greater anxiety and depressive symptoms and everyday discrimination (*P* values of .006, .032, and .007, respectively) compared with those who returned at least one biospecimen sample. The missing sample group did not differ by sex or other characteristics (Table S1 in Multimedia Appendix 1). ADI was greater for 5 participants with inverse CDR compared with those with physiologically congruent CDR (8.2, SD 2.5 vs 5.03, SD 2.5, *P*=.01), but other descriptive characteristics of these 5 individuals did not differ from those of the 32 other participants (Table S2 in Multimedia Appendix 1).

### Exploratory Analysis

**Figure 3:**
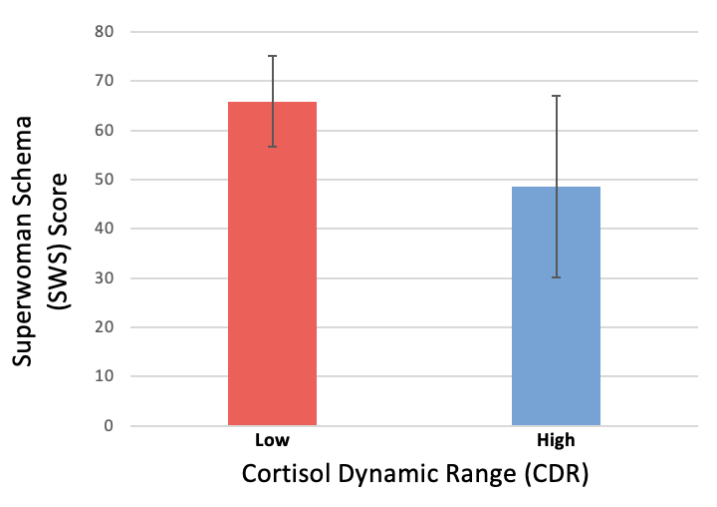
Superwomen schema score by low and high cortisol dynamic range (CDR) groups. The graph displays mean Superwoman Schema (SWS) score by low versus high CDR dichotomized at the median (1.29). Compared with those with high CDR (most dynamic), those with low CDR (least dynamic) had significantly greater SWS scores (range 0-105) with higher scores indicating greater endorsement of SWS characteristics, or greater burden of high effort coping (mean SWS score 65.9 [SD 9.1] for low CDR group vs mean SWS score 48.6 [SD 18.4] for high CDR group, *P*=.03).

Median overall CDR was 1.4 (SD 0.74), with a mean value of 0.78 and 1.99 for below and above the median, respectively. Demographic characteristics did not differ for individuals in the low and high CDR groups (Table S3 in Multimedia Appendix 1). No differences in demographic characteristics or study variables were observed by sex (Table S4 in Multimedia Appendix 1). In African American women, a higher SWS score was observed in individuals with lower CDR (*P*=.03; Figure 3).

Since no demographic characteristics differed when they were compared by (1) high versus low CDR group, (2) inverse CDR versus physiologic CDR, or (3) sex, only unadjusted models were used in exploratory regression analyses. In African American women, perceived stress and anxiety, but not depressive symptoms nor sleep quality, were significantly associated with lower CDR (GPSS: *β*=–0.07, *P*=.01, anxiety: *β*=–0.06, *P*=.01; Table 2). Greater SWS and greater distance from George Floyd Square were also associated with lower (less dynamic) CDR (*β*=–0.02, *P*=.04, and *β*=–0.02; *P*=.01), but no association was observed with ADI. No associations were seen with CDR for men, but analyses were limited by small sample size (n=12, Table S5 in Multimedia Appendix 1). Of note, analysis also demonstrated a trend toward an association between suboptimal participant-reported sleep quality and lower CDR in African American women (*P*=.05).

**Table 2:** Linear regression analyses of chronic stress exposures on cortisol dynamic range outcome in the Fostering African-American Improvement in Total Health! (FAITH!) Heart Health+ study for African American women.

Independent variable (exposure)		β coefficient	*P* value	95% CI
**Individual**				
	Psychosocial stress (GPSS^a^)	–0.07^b^	.01	–0.11 to –0.02
	Anxiety symptoms	–0.06^c^	.01	–0.11 to –0.01
	Depressive symptoms	–0.04	.27	–0.11 to 0.03
	Sleep quality (PSQI^d^)	–0.07^e^	.05	–0.13 to 0.00002
	High-effort coping: Superwoman Schema (SWS)	–0.02^f^	.04	–0.03 to –0.0008
**Interpersonal**				
	Everyday discrimination	0.01	.74	–0.32 to 0.04
**Structural**				
	Area deprivation (ADI)^g^	0.08	.17	–0.04 to 0.19
	Distance from George Floyd Square (km)^h^	–0.02 (higher distance, lower CDR^i^)	.01	–0.03 to –0.004

^a^GPSS: Global Perceived Stress Scale.

^b^Higher GPSS, lower cortisol dynamic range (CDR).

^c^Higher anxiety, lower CDR.

^d^PSQI: Pittsburgh Sleep Quality Index.

^e^Higher PSQI (worse sleep quality), lower CDR.

^f^Higher SWS, lower CDR.

^g^ADI: Area Deprivation Index.

^h^Adjustment for income did not change the results (β=–0.02, *P*=.02, 95% CI –0.03 to –0.003).

^i^To lend to interpretability of the results as related to distance from George Floyd Square, exploratory analysis revealed that closer proximity to George Floyd Square was associated with lower income (7 out of 9 African American women with annual income of <US $50,000 who lived closer, compared with 7 out of 8 African American women with income ≥US $50,000 who lived further away, *P*=.007). As per exploratory analysis, a trend (*P*<.10) was observed for further distance from George Floyd Square as associated with greater stress (mean GPSS 5.1, SD 4.1 vs mean GPSS 9.9, SD 6.2, *P*=.07) and greater SWS score (mean 48.6, SD 17.1 vs mean 62.8, SD 16.4, *P*=.08). The association between distance from George Floyd Square and CDR remained after adjusting for income (Table S5 in Multimedia Appendix 1).

## Discussion

### Principal Findings

**Figure 4:**
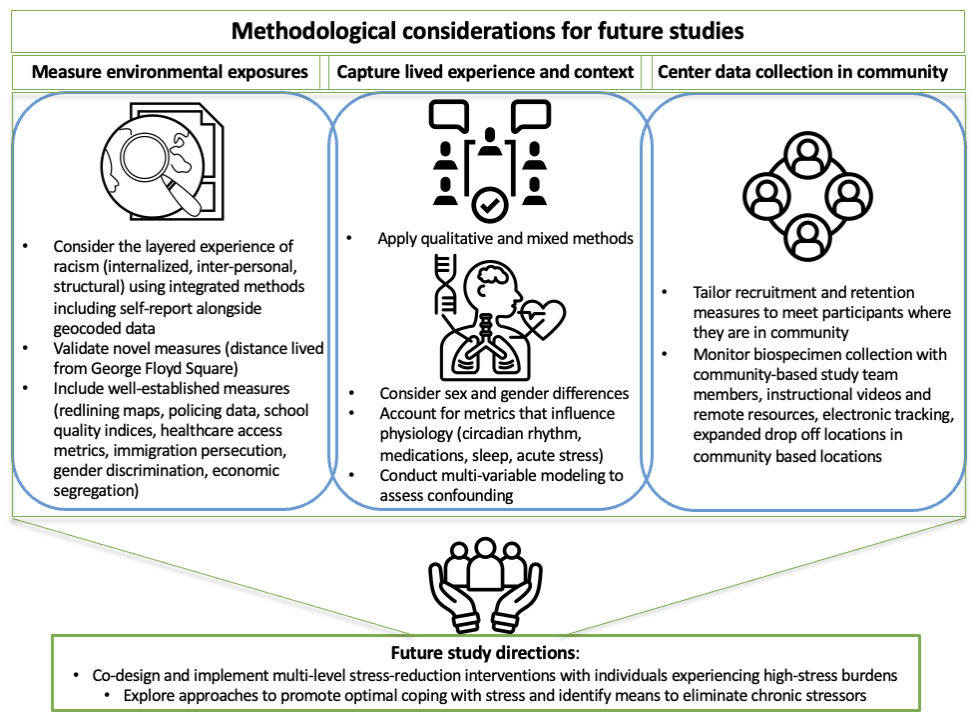
Methodological considerations and future directions. The figure demonstrates an integration of lessons learned and future directions to replicate and expand on the exploratory findings of this study using expanded quantitative data to capture measures of structural racism, including data exploring lived experience and context of perspectives as well as physiological impacts (eg, influences on cortisol and stress physiology), and use community-engaged methods for data collection, including biospecimen collection. Such methodological considerations are posed with the aim of adapting and building supports to directly and indirectly address minority stress and related consequences to health [9].

In this CBPR study assessing multilevel, psychosocial influences on CDR within a community-based sample of African American individuals, we found that the measurement of CDR using 2 timepoints of participant-collected and returned biospecimen samples for cortisol analysis was feasible with 70% of consented participants having a CDR measurement in the study, which is above our hypothesis of high engagement above 50% as compared with prior literature on studies collecting saliva biospecimens [50]. Though fewer consented participants (≈60%) had usable samples for the herein exploratory analysis, this is still also above the hypothesis of 50%. Additionally, our study sample demonstrated hypothesis-generating, but significant associations between stress, high effort coping, and environmental factors with CDR in African American women. Overall, our findings highlight the need for further studies to consider the potential of CDR as a biomarker of toxic stress in community-engaged health equity research studies, including multimodal data collection with the aim of replicating and confirming the current findings in larger cohorts. Further, given the potential implications of our findings, it will be important to understand this and related work through the use of refined community-engaged qualitative and mixed methods, including intentional attempts to capture the lived experiences of racism in greater depth (Figure 4), as we have achieved in the FAITH! HH+ ancillary study and prior work [31,51]. Multilevel modeling can also be used to model multilevel variables simultaneously to delineate pathways. Our study aimed to explore the impacts of racism on health, hypothesized to manifest as impacts on stress physiology, which we discuss is a crucial approach to health equity work. The remainder of our discussion will focus on contextualizing our findings of an association between coping and CDR in Black women and sex-based differences in stress and stress physiology. We then shift to commentary on the unique methodology of this study, which incorporated geocoded variables and feasibility data around the use of CDR.

To advance health equity work, community-engaged research should aim to measure exposures and outcomes with methods that account for multiple levels of influence on health outcomes [5]. As racism is multifactorial, occurring at individual, interpersonal, and structural levels, studies of the impact of racism in marginalized communities require a multimodal measurement approach. Survey methods allow for self-report on stress and coping, and experiences of interpersonal racism. Geospatial methods allow for the assessment of the impact of racism and discrimination on the environment. Understanding the mechanisms underpinning the impact of racism may offer future studies the ability to assess robust interventions that may mitigate such effects, though our study also brings attention to the need for such interventions to be intentionally contextually and culturally adapted. That said, to assess the impact of racism at all levels on health, an outcome measure that assesses physiological toxic stress is needed. Prior studies have suggested that CDR is ideal for such assessment in community-engaged research [5].

Our main exploratory study result demonstrated noteworthy findings related to the relationship between different levels of psychosocial stress in African American women, from personal to community/environmental-level, and physiologic toxic stress, measured through CDR. Specifically, in regression analyses, we demonstrated that greater perceived stress, anxiety, SWS score (high effort coping), and living distance to George Floyd Square were associated with biological dysregulation of diurnal cortisol, measured herein as lower (less dynamic) CDR among African American women participants. Notably, we did not observe a relationship between interpersonal racism events (everyday discrimination) and CDR. These findings taken together may reflect how the perception and coping manifestations of stress are more proximate to the stress response than events. This may be supported by a theorized framework of the Substance Abuse and Mental Health Services Administration of impressionably stressful or traumatic experiences described as the “three E’s”—event, experience, and effect [52]. Events of discrimination may be individually or collectively manifested into an experience of stress at the personal level, as sometimes described as internalized racism [53]. While more research is needed to support this concept, it is possible that such experiences then underpin effects on physiology, as measured by lower or less CDR. This is evidenced by a study of African American adults (N=312, majority female [≈55%]) that demonstrated anxiety symptoms as a mediator of the relationship between everyday discrimination and nondiurnal cortisol (random time of collection by saliva) [54], and thereby anxiety symptoms as more proximate on the potential mechanistic pathway to physiological (cortisol) dysregulation than the everyday events of discrimination. Importantly, this same study observed that only anxiety symptoms and not depressive symptoms were a significant mediator, which corroborates the findings in our study of a relationship between anxiety but not depressive symptoms and CDR. In addition to anxiety, the experience of stress and racism may be more directly related to an individual’s manifestation of coping [28]. With that, as hypothesized, this study observed the manifestation of the SWS (high effort coping specific to African American women) in association with biological dysregulation of cortisol (CDR), despite the fact that everyday discrimination was not. Last, though we followed previously described procedures to use the Everyday Discrimination Scale to score a mean value of exposure to everyday discrimination [41,55], it is important to note that standardization of the scoring of the Everyday Discrimination Scale has varied in the literature [56]. This may have an impact on the precision of its measurement, depending on the population evaluated. Still, our study is unique given its inclusion of measurements that account for different layers of exposure to racism and discrimination, perhaps not captured by the Everyday Discrimination Scale. Future studies should be powered to assess the applications of different measures of stress and racism on a population-wide scale, from questionnaires like the Everyday Discrimination Scale to biological and environmental measures, as we included herein.

The findings we described in Black women in our study must be contextualized amid the literature, which demonstrates sex-based differences in the manifestations of stress. We observed an association between experiences of stress and toxic stress (CDR) in participants who identified as African American women, but not in African American men, which is consistent with the literature, which suggests experiences of, and responses to, racism and stress differ between African American women and men. The SWS posits that African American women display strength, resistance, determination, and selflessness in the face of significant stress and challenge, which has been understood through the perspectives of African American women as necessary for preservation of self and family or community [39]. However, the views of African American women of the SWS also reveal a common theme of the potential consequences of this form of high effort coping, inclusive of stress-related health behaviors, and stress embodiment [39]. Further, the literature has described that flatter diurnal cortisol reactivity may occur for African American women, compared with African American men. In one study, salivary cortisol samples were collected before and after a highly publicized accusation of campus gender-based violence and rape that was particularly associated with high stress for African American women on campus, in both African American women and men [16]. This study found that results differed by sex, where women had lower cortisol levels on average before the event, but demonstrated higher cortisol after the event compared with men. Another study assessed stress responsiveness, including change in cortisol levels after a writing task where African American participants wrote about experienced justice and injustice. In this study, African American women demonstrated lower cortisol recovery after the stress-inducing task [57], suggesting a less dynamic cortisol response associated with stress, consistent with our observations. While another study did not find differences in analyses of the relationship among discrimination, anxiety, and cortisol levels by sex, it was described by such authors that much literature has not explored the moderation of sex, while most often it was only controlled for as a variable [54]. Perhaps due to the small sample size, we did not observe an association between CDR and psychosocial stress or coping in African American men. Therefore, it remains to be explored if an association may be observed between high effort coping in African American men (eg, John Henryism) and CDR. Notably, it has been observed that sex differences exist across cortisol measures and studies, but findings differ by method of collection (eg, urine, saliva, serum, and epigenetic) and context (eg, with or without an acute stress exposure, stressor types, and different collection times) [58,59]. Therefore, in the design of future studies, investigators must consider sex-based differences and contexts, but alongside cortisol measurement methodology.

Beyond the exploratory findings, our study also highlights some methodological considerations that could inform future work related to the inclusion of geocoded variables as well as the inclusion of feasibility data around the use of the CDR measure itself. As it relates to the geocoded variables included, this study found an association between a greater distance from George Floyd Square in Minneapolis and less dynamic CDR in African American women. George Floyd Square in Minneapolis is the site of the murder of Mr George Floyd and, therefore, it is a locus for social unrest, which also has historically been a neighborhood marked by marginalization and economic disenfranchisement [60]. Prior literature may contextualize these findings. For example, studies have demonstrated that populations more removed from socioeconomic hardship, or more proximate to opportunity for socioeconomic mobility, have greater levels of reported perceptions of racism and discrimination, thus greater potential stress and related signs of physiological toxic stress [61,62]. It is proposed that African American individuals of higher socioeconomic status, which equates to living in areas with greater social and economic opportunity and resources (as may be the case for individuals living further from George Floyd Square), may live in more integrated geographic settings, therein posing greater exposure to experienced and internalized racial discrimination and stress [63]. Despite the proximity to opportunity, inequity exists in social and economic gains afforded to racialized groups [64]. Importantly, though there is potential conflation between socioeconomic disadvantage and racism in using the proximity to George Floyd Square as a proxy measure for structural racism, as may also be the case for the use of the ADI. Accordingly, still more research is needed to understand the use of our indicators in intentionally integrating the context of the event of Floyd’s death and socioeconomic factors in measuring structural racism. Other hypotheses could explain the results we observed. For one, it is also possible that people who live close to the square have physiologically adapted to the acute stress of his death in the setting of a strong community of support, or, alternatively, were not physiologically impacted by the sudden additional stressor in the setting of long-standing marginalization and toxic stress—either potentially resulting in a lack of observed association with CDR at the time of this cross-sectional ancillary study, whereas, those that live further away may be experiencing the ongoing impacts of the stress of Floyd’s death to a greater degree at the time of this study, having not yet adapted.

Our study also aimed to assess the feasibility of measuring CDR in a community-engaged cohort. The first step before measurement involved assessing the feasibility of collection of the salivary samples, and this is the main part of the feasibility data we can assess in the context of the literature, given that fewer studies have specifically yet assessed CDR. Our result of 70% participation in biospecimen sample (saliva) collection is consistent with existing literature when multiple contextual studies are taken together, potentially even indicating a high engagement rate for participant-dependent sample collection in a cohort of African American individuals. For example, in a community-based study of various types of biospecimen collection (stool, urine, and hair), samples were returned for 65% of participants (range 60.3%-65.6%) [65]. Notably, participants who identified as African American returned 10% fewer baseline study samples than participants who identified as White, and were 20% less likely to return samples at 2 time points [65]. The literature has described barriers to biospecimen participation in African American populations, including perceptions of mistrust in research influenced by a history of unjust medical and research practices [66,67]. With these factors considered, the participation of 70% of participants in biospecimen collection and return within our sample appears adequate and even remarkable, in the face of challenges for studies in marginalized communities amid the COVID-19 pandemic—a time of high competing demands. Notably, despite this promising result, this percentage was lower than reported participation in a prior similar cohort of African American participants where community-based study team members in the field collected biospecimens for CDR assessment directly from participants’ in their homes and neighborhoods in >90% of participants [5]. Differences in characteristics of individuals in this study who did not return biospecimen samples may inform reasons for this lower return rate. Those missing samples were younger, with greater mood symptoms, and reported greater experiences of everyday discrimination than those returning samples. It is possible that the competing priorities of young adulthood, such as work and supporting families, may pose challenges to study participation, especially for sample collection at the times of day when one may be preparing for the day (morning) or involved in events like school or work (late afternoon). Further, those with greater anxiety and experiences of discrimination may have fears or hesitations that are generalized or related to trust in research that pose a challenge to participation [68,69]. Studies may direct resources for enhanced study team-participant engagement in subpopulations facing such challenges. Direct interaction and communication with study team members at the time of sample provision may offer an added sense of transparency to improve African American engagement in biospecimen collection in research and biobanking [66]. Still, our data are consistent with the literature, further corroborating the feasibility with external validity of CDR assessment as CDR in our cohort was within the range of prior studies, but slightly below the mean of those studies (1.3 for the current compared with 1.7-1.9 of prior) [5,6,8]. This may indicate a higher toxic stress burden (lower or less dynamic CDR) overall in our cohort. Most importantly, our results in the context of the salivary cortisol collection feasibility evidence in the literature demonstrate that studies implementing fewer collection points for measurement (as only 2 are required for CDR), may be more feasible in community contexts. Still, more research is needed to explore and validate this methodology in populations experiencing socioeconomic disadvantage.

When including cortisol analyses in research, one must consider influences on the diurnal context, namely, sleep. Our study uniquely measured sleep quality and its association with CDR, which is a crucial consideration in community-engaged studies with African American populations, given the significant impact sleep has on physiological diurnal cortisol responses and how exposure to discrimination can impact sleep quality [5,70]. Our results, though limited by missing data for the sleep quality measure, did demonstrate a trend toward an association between suboptimal participant-reported sleep quality and lower CDR in African American women (*P*=.05), which is consistent with the direction that would be physiologically hypothesized. We also observed 5 participants with CDR that was inverse or opposite of what the predicted physiological cortisol points would be (instead demonstrating morning nadir and later afternoon peak of diurnal cortisol), which may reflect their sleep-wake cycles being reversed, though this data was not known in this study. It is possible that participants facing greater socioeconomic hardship also face disruptions to sleep-wake cycles and could particularly benefit from personalized study team support in sample data collection to align with measures of time of collection and sleep metrics. Future studies should aim to explore aspects of “in-community” methodology that could be leveraged to enhance participant-led return of biospecimen samples with accompanying details of sleep timing and quality data.

### Strengths and Limitations

To the best of our knowledge, this study is among the first to assess the relationship between layers of exposure to racism and toxic stress by CDR in a community-engaged sample. Therefore, a major strength of the study is the community-informed and participatory nature of the study design and data collection. Further, the geographic location and timing of our study were unique, as Minnesota was an epicenter of social unrest and the location of a particular time of extreme stress for African Americans after the death of Mr George Floyd. While prior studies have predominantly considered sex as a covariate, a strength of our study was its stratification by sex. Our study also reported feasibility outcomes to inform the design of CDR collection and measurement in future community-engaged studies.

There are several limitations to this study. There were differences in consented individuals who did and did not return biospecimen samples. However, this insight offers opportunities to enhance feasibility to extended communities and populations who otherwise may be less represented in community-engaged research. Importantly, cortisol, while a useful and feasible biomarker, is influenced by numerous factors (eg, circadian rhythm, medications, sleep, and acute stress). Relatedly, there were no exclusion criteria for factors that may impact cortisol levels, such as night shift working or the use of medications (eg, steroids or beta-blockers). Accordingly, future studies should aim to assess pharmacotherapies and other health factors that may impact cortisol dynamics. Most importantly, related to the broader implications of our work, it must be stated that cortisol alone cannot fully capture toxic stress or systemic racism’s complex physiological footprint. Therefore, multimodal approaches incorporating complementary biological and psychosocial metrics should be used in future research. Still, when a study does aim to include cortisol as part of this multimodal approach, we herein provide evidence for the feasibility of CDR as a measure to include. In doing so, investigators must still consider the limitations of its inclusion.

While CDR requires only 2 time points, making it less dependent on variations in collection timing than other cortisol measures, our results must be interpreted in the context that CDR measurement is dependent on nadir and peak in diurnal cortisol. Thus, the collection of only 2 time points was a robust attempt to align with an estimation of the time of day when nadir and peak may occur, which is an estimation of what precise nadir and peak may be. Our study methods offered a broad collection window (2 hours), while a peak at awakening response occurs over a shorter period (≈45 minutes), suggesting a source of deviation from ideal measurement and the introduction of potential collection and measurement error [71]. Accordingly, results assessing associations with CDR must be interpreted with caution. However, it is notable that the exposures of perceived stress, high effort coping, and socioeconomic hardship did not differ in the missing compared with the non-missing group, which supports the interpretability of the association between these variables and CDR reported. While our study had lower biospecimen participation rates than prior studies, we demonstrated the feasibility of CDR measurement with just 2 timepoints of salivary cortisol measured.

Although 5 participants had inverse CDRs from what would be physiologically predicted, their descriptive characteristics, including self-reported sleep quality measures, did not differ from those of others in the cohort. It is possible that such inverted measures may be due to another limitation of our study—the idea that self-collection without monitoring could introduce erroneous or misclassified samples. Therefore, self-collection, while a strength for enhancing community-engaged metrics in research, may also have to be balanced with addressing the limitations of data collected without the support of an algorithmic procedure or research team. Future studies may consider how different methodologies of collection could strengthen the data and contribute to addressing these potential discrepancies, such as engaging community health workers or research team members in the community, using remote resources like instructional videos, using electronic time stamping through QR code use or a dedicated smartphone app, and providing a variety of convenient community biospecimen drop-off locations to study team (eg, churches, salons, and barbershops).

Beyond CDR, specifically, our study was notable for limitations in our data availability. The limitations of the CDR measurement and the data availability and sample size led us to highlight that this was an exploratory analysis and, accordingly, remain unadjusted, which leaves the possibility of confounding in our findings. Future powered studies should consider multivariable modeling to ensure assessment for confounding. For example, due in part to missingness, our study had a small sample size for participants with complete cortisol and exposure variable measures. The small sample size led to demographic skew toward older individuals, more female participants, and potential attrition bias toward less stressed participants who returned specimens. Our study was not adequately powered to conduct additional moderation/mediation analyses by sex. These limitations affect generalizability and the likelihood that those most burdened by stress may be underrepresented. It is possible that this skewed our findings toward the mean, and true findings may have been larger or inclusive of men, for example. Future studies should use tailored recruitment and retention strategies to engage younger and more distressed individuals. Additionally, the data used for this study aimed to include measures to interpersonal and environmental discrimination (every day discrimination, and the geographical indicators including ADI and proximity to George Floyd Square); however, the lack of additional validated measures of structural racism may risk bias for failing to observe confounding observations in the relationship between stress and biological outcomes such as other forms of social or neighborhood disadvantage rather than racism, per se. Specifically, the geographic indicators used in this study are imperfect proxies for structural racism that may not fully capture systemic racism, which is multifaceted and structural across domains. Future studies may consider the addition of measures, including those validated for capturing historical and structural racism, such as redlining maps, policing data, school quality indices, health care access metrics, immigration persecution, sex-based discrimination, or economic segregation indices, to deepen understanding of the impacts of historical and structural racism. Still further, future studies should therefore aim to evaluate the relationship between self-reported health and coping with CDR in individuals from socially disadvantaged but nonracially marginalized groups, or from racially diverse groups experiencing violence or trauma. Nonetheless, our exploratory analyses demonstrated findings consistent with our hypotheses of the relationship between stress, racism, and CDR, warranting further consideration in larger-scale community-engaged studies.

### Call to Action

Our study demonstrates how experiences of racism, even if not perceived and self-reported as overt discrimination, may manifest in impacts on the stress coping associated with physiological manifestations. Recognizing systemic racism as deeply embedded in societal structures—not merely the product of individual actions or discriminatory experiences—allows us to reframe the issue from what some may consider as blame to one of collective action. Each of us can play a role in dismantling these systemic barriers by educating ourselves, advocating for equitable policies, supporting community initiatives, and integrating equity-centered approaches in research and practice. Through such shared efforts, we can contribute to reducing the toxic stress and health disparities that disproportionately impact marginalized populations. At the policy level, actions may include critically examining and reforming institutional policies or research practices that perpetuate inequities and advocating for equitable resource allocation and amplifying the leadership and voices of marginalized communities at local, state, and national levels. At the community level, this may involve engaging community partnerships in all aspects of work and ensuring that the voices of those represented are involved in all steps of the processes for projects and interventions. At the interpersonal level, this may include the incorporation of trauma-informed practices and care. For researchers seeking to advance this area of work, our study demonstrates both a feasible approach to community-engaged research that aims to integrate biological constructs and mechanistic explorations, while also suggesting that future work should aim to develop programs and interventions to support Black women in off-setting stress burden and optimizing coping in the face of stress and responsibilities.

The FAITH! HH+ ancillary study aims will take the next steps toward actionable strategies to improve community health. As a CBPR initiative, the overarching FAITH! program approach extends beyond community-led biospecimen collection to include structured processes for data sharing and co-interpretation with community partners, ensuring that findings are contextualized and aligned with community priorities. Insights from this study directly inform the iterative development and evaluation of culturally tailored interventions, most notably the FAITH! App, a mobile lifestyle intervention shown to improve cardiovascular health metrics among African American adults and currently undergoing continued community-informed optimization [30,72]. In addition, FAITH! is implementing educational strategies that address the psychosocial impacts of structural racism and chronic stress [35], including social support [73], faith-based health messaging [74], and interventions to simultaneously improve digital health literacy as well as cardiovascular and mental health outcomes [75]. Collectively, these efforts represent a deliberate shift from observational research toward the cocreation of actionable, community-centered solutions that support sustained improvements in population health outcomes.

### Conclusions

Our study demonstrates that the measurement of CDR through participant biospecimen collection is feasible within a community-based study of community-dwelling African Americans. Potential challenges to overcome in future research include providing enhanced resources to support participant biospecimen collection. Our study findings suggest that CDR is a potentially feasible measure of chronic stress physiological burden (eg, toxic stress), given the association between subjective (participant-reported) and objective (geospatial) measures of exposure to psychosocial and socioeconomic stress and lower diurnal cortisol in African American women. Taken together, our study provides evidence that the collection of multimodal (survey, environmental, and physiologic) data in community-engaged research is necessary to investigate the impact of racism at the individual, interpersonal, and structural levels. Further, CDR may serve as a biomarker in community-based studies aiming to assess the effectiveness of multilevel interventions or strategies at a physiologic and mechanistic level in historically marginalized communities most impacted by health disparities.

## Appendix

Multimedia Appendix 1 Additional tables and figures.

## Abbreviations

ADI: Area Deprivation Index
CBPR: community-based participatory research partnership
CDR: cortisol dynamic range
CES-D: Center for Epidemiological Studies Depression
FAITH!: Fostering African-American Improvement in Total Health!
GPSS: Global Perceived Stress Scale
HH+: Heart Health+
HPA: hypothalamic-pituitary-adrenal
NIMHD: National Institute on Minority Health and Health Disparities
PROMIS: Patient-Reported Outcomes Measurement Information System
SWS: Superwoman Schema
