# Supplementary material for: A Community-Engaged Approach for Assessment of Cortisol Dynamic Range and Multilevel Chronic Stress in African Americans: FAITH! Heart Health+ Ancillary Study
Source: J Particip Med. 2025 Dec 1;17:e69604. doi: 10.2196/69604 (PMC12706454; doi:10.2196/69604)
Supplement: Multimedia Appendix 1 [file jopm_v17i1e69604_app1.docx]

**SUPPLEMENTAL MATERIAL**

**Exploration of Cortisol Dynamic Range as a Biomarker of Individual, Interpersonal, and Structural Chronic Stress in African Americans: FAITH! Heart Health+ Ancillary Study**

This supplement contains the following elements:

**Figure S1.** Flyer for Community-Engaged Recruitment into the FAITH! Heart Health+ Study

**Table S1.** Characteristics of FAITH! Heart Health+ Study Participants by Complete Cortisol versus Missing Cortisol Data

**Table S2.** Sociodemographic Characteristics, Chronic Stress Measures and Cortisol Dynamic Range of FAITH! Heart Health+ Study Participants by Physiologically Consistent versus Inverse Cortisol Dynamic Range

**Table S3.** Sociodemographic Characteristics of FAITH! Heart Health+ Study Participants by Cortisol Dynamic Range

**Table S4.** Characteristics, Chronic Stress Measures and Cortisol Dynamic Range of FAITH! Heart Health+ Study Participants by Sex

**Table S5.** Linear Regression Analyses of Stressful Exposures on Cortisol Dynamic Range Outcome (continuous) of African American Men in the FAITH! Heart Health+ Study

**Figure S1.** Flyer for Community-Engaged Recruitment into the FAITH! Heart Health+ Study

**
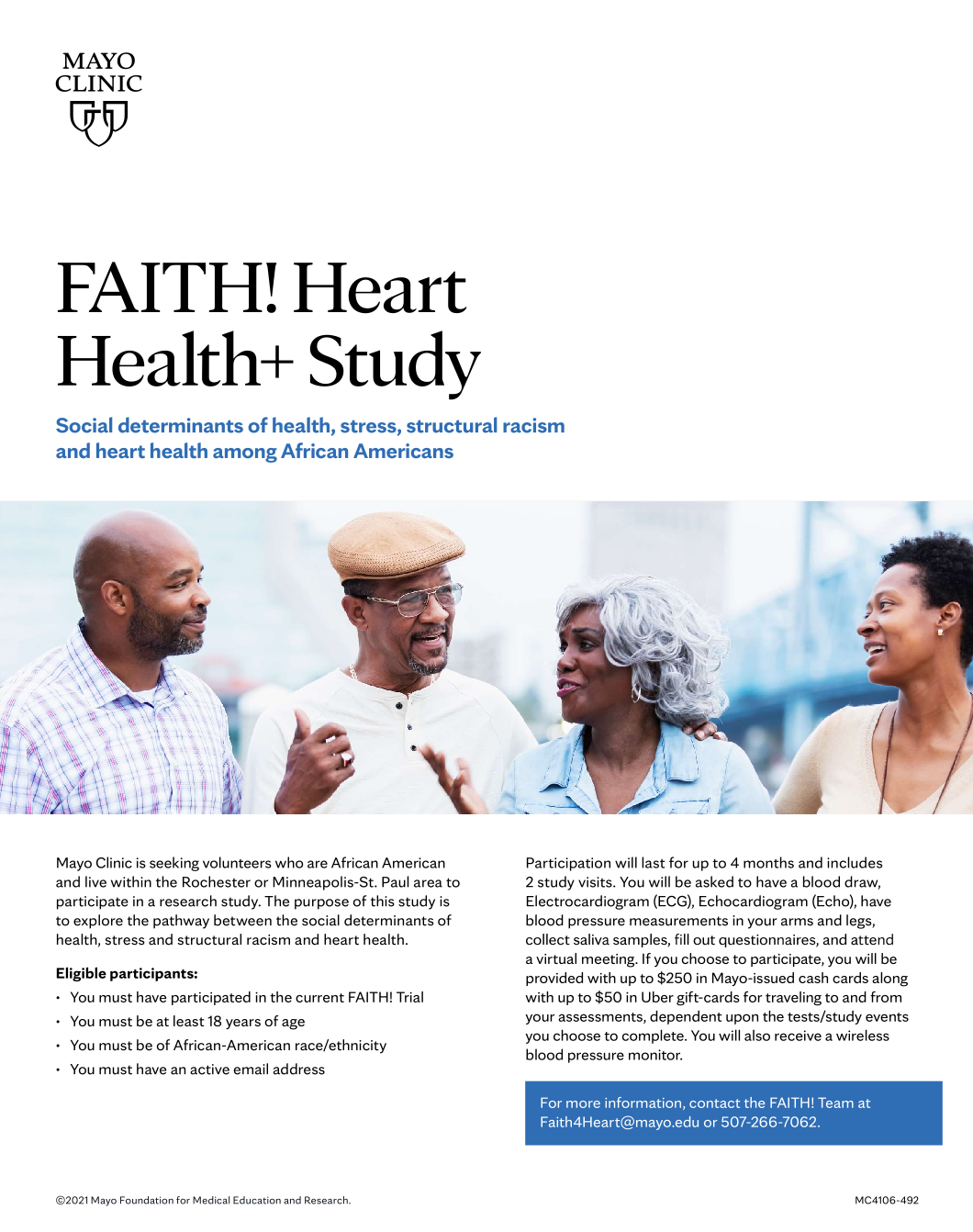
**

**Table S1.** Characteristics of FAITH! Heart Health+ Study Participants by Missing Cortisol vs. Complete Cortisol Data

|  | Cortisol  n=37 | Missing  n=19 | Total (n=58) | *P*-value |
| --- | --- | --- | --- | --- |
| **Demographic Characteristics** | | | | |
| **Sex**, n |  |  | n=56 | 0.45 |
| Men | 13 | 5 | 18 |  |
| Women | 24 | 14 | 38 |  |
| **Age**, years | 57.5 (10.9) | 48.9 (12.1) | 54.6 (11.95) | **0.009** |
| **Annual Household Income**, n |  |  | n=56 | 0.80 |
| <$50,000 | 12 | 7 | 19 |  |
| ≥$50,000 | 20 | 10 | 30 |  |
| **Education**, n |  |  | n=54 | 0.74 |
| ≤High School | 3 | 2 | 5 |  |
| >High School | 33 | 16 | 49 |  |
| **Variables** | | | | |
| ***Individual*** | | | | |
| **Psychosocial stress (GPSS)** | 6.4 (4.8)  n=36 | 8.4 (5.4)  n=17 | 7.1 (5.0) n=53 | 0.18 |
| **Anxiety symptoms** | 11.3 (5.4)  n=35 | 16.0 (6.1)  n=19 | 12.98 (6.1) n=54 | **0.006** |
| **Depressive symptoms** | 15.8 (5.0) n=37 | 19.7 (8.0) n=18 | 17.05 (6.3) n=55 | **0.032** |
| **Sleep Quality (PSQI)** | 6.3 (4.2)  n=29 | 9.2 (5.8) n=12 | 7.15 (4.9) n=41 | 0.09 |
| **High effort coping: Superwoman Schema (women only)** | 57.7 (17.09) n=24 | 66.6 (19.6) n=15 | 61.1 (18.4) n=39 | 0.14 |
| **High effort coping: John Henryism** | 48.3 (5.8) n=37 | 46.9 (6.7) n=18 | 47.8 (6.1) n=55 | 0.43 |
| ***Interpersonal*** | | | | |
| **Discrimination** | 22.0 (7.5)  n=38 | 27.8 (7.1)  n=19 | 24.0 (7.8) n=57 | **0.007** |
| ***Structural*** | | | | |
| **Area Deprivation (ADI)** | 5.5 (2.6) n=37 | 5.7 (2.7) n=20) | 5.5 (2.7) n=57 | 0.75 |
| **Distance George Floyd Square**, km | 20.56 (32.2) n=37 | 30.8 (44.6) n=20 | 24.2 (4.9) n=57 | 0.33 |

Total sample size of n=58 consented for any biospecimen sample collection; n=56 with demographic data available for comparison (eg, 37 with cortisol and 19 missing cortisol for a total of N=56); N=53 consented specifically to saliva collection and received kits by mail.

All values mean (standard deviation, SD) unless otherwise specified.

ADI = Area Deprivation Index; GPSS = Global Perceived Stress Scale; PSQI = Pittsburgh Sleep Quality Index.

**Table S2.** Characteristics of FAITH! Heart Health+ Study Participants by Physiologically Consistent vs. Inverse Cortisol Dynamic Range (CDR)

|  | Physiologic CDR  n=32 | Inverse  CDR  n=5 | Total (n=37) | *P*-value |
| --- | --- | --- | --- | --- |
| **CDR** | 1.4 (0.7) | 1.3 (0.5) | 1.4 (0.8) | 0.73 |
| **Demographic Characteristics** | | | | |
| **Sex**, n |  |  |  | 0.50 |
| Men | 11 | 1 | 12 |  |
| Women | 20 | 4 | 24 |  |
| **Age**, years | 57.5 (11.6) | 56.8 (7.7) | 54.6 (12.0) | 0.90 |
| **Body Mass Index**, kg/m^2^ | 36.2 (8.1) n=31 | 31.1 (4.2) n=5 | 35.5 (7.9) n=36 | 0.18 |
| **Annual Household Income**, n |  |  |  | 0.05 |
| <$50,000 | 12 | 0 | 12 |  |
| ≥$50,000 | 14 | 5 | 19 |  |
| **Education**, n |  |  |  | 0.46 |
| ≤High School | 3 | 0 | 3 |  |
| >High School | 37 | 5 | 32 |  |
| **Variables** | | | | |
| ***Individual*** | | | | |
| **Psychosocial stress (GPSS)** | 6.5 (5.1)  n=30 | 5.8 (3.9)  n=5 | 6.4 (4.9)  n=35 | 0.80 |
| **Anxiety symptoms** | 11.0 (5.5)  n=29 | 13.8 (5.3) n=5 | 11.4 (5.5) n=34 | 0.31 |
| **Depressive symptoms** | 15.3 (4.4)  n=31 | 18.6 (8.1)  n=5 | 15.8 (5.0) n=36 | 0.18 |
| **Sleep Quality (PSQI)** | 6.36 (4.4)  N=25 | 7.3 (2.1)  N=3 | 6.5 (4.2) n=28 | 0.72 |
| **High effort coping: Superwoman Schema (women only)** | 55.5 (17.3)  N=20 | 68.5 (11.6)  N=4 | 57.7 (17.1) n=24 | 0.17 |
| **High effort coping: John Henry** | 48.0 (6.1) n=31 | 48.6 (2.8) n=5 | 48.1 (5.7) n=36 | 0.83 |
| ***Interpersonal*** | | | | |
| **Discrimination** | 21.8 (7.5)  n=32 | 24.4 (7.0)  n=5 | 22.2 (7.5) n=37 | 0.48 |
| ***Structural*** | | | | |
| **Area Deprivation (ADI)** | 5.0 (2.5) n=31 | 8.2 (2.5) n=5 | 5.5 (2.7) n=36 | **0.012** |
| **Distance George Floyd Square**, km | 23.5 (34.5) n=31 | 6.0 (4.8) n=5 | 45.9 (21.6) n=36 | 0.27 |

All values mean (standard deviation, SD) unless otherwise specified.

ADI = Area Deprivation Index; GPSS = Global Perceived Stress Scale; PSQI = Pittsburgh Sleep Quality Index.

**Table S3.** Sociodemographic Characteristics of FAITH! Heart Health+ Study Participants by Cortisol Dynamic Range (CDR)

|  | Low CDR  n=16 | High CDR  n=16 | Total (n=32) | *P*-value |
| --- | --- | --- | --- | --- |
| **CDR** | 0.8 (0.4) | 2.0 (0.4) | 1.39 (0.7)  [min,max] [0.02,2.8] |  |
| **Demographic Characteristics** | | | | |
| **Sex**, n |  |  | n=31 |  |
| Men | 8 | 4 | 12 | 0.14 |
| Women | 8 | 12 | 20 |  |
| **Body Mass Index**,  kg/m^2^ | 38.0 (8.2)  n=15 | 34.5 (7.9) n=16 | 36.2 (8.1)  n=31 | 0.23 |
| **Age**, years | 56.4 (13.2) | 58.5 (10.2) | 57.5 (11.6)  n=31  [min,max] [30,86] | 0.63 |
| **Annual Household Income**, n |  |  | n=31 | 0.67 |
| <$50,000 | 5 | 7 | 12 |  |
| ≥$50,000 | 7 | 7 | 14 |  |
| **Education**, n***** |  |  | n=30 | 0.46 |
| ≤High School | 2 | 1 | 3 |  |
| >High School | 12 | 15 | 27 |  |

All values mean (standard deviation, SD) unless otherwise specified.

*No significant difference in comparing those with some college vs. college degree or more.

**Table S4.** Characteristics of FAITH! Heart Health+ Study Participants by Sex

| **Sex**, n | **Men**  n=12 | **Women**  n=20 | Total  n=32 |  |
| --- | --- | --- | --- | --- |
| **CDR** | 1.1 (0.9) n=12 | 1.5 (0.6)  n=20 | 1.39 (0.7)  n=32 | 0.13 |
| **Demographic Characteristics** | | | | |
| **Age**, years | 55.5 (14.4) n=11 | 58.6 (10.0) n=20 | 57.5 (11.5)  n=31 | 0.50 |
| **Body Mass Index**, kg/m^2^ | 36.1 (8.6) n=11 | 36.2 (8.1)  n=20 | 36.2 (8.1)  n=31 | 0.98 |
| **Annual Household Income**, n |  |  |  | 0.56 |
| <$50,000 | 3 | 9 | 12 |  |
| ≥$50,000 | 5 | 9 | 14 |  |
| **Education**, n |  |  |  | 0.26 |
| ≤High School | 2 | 1 | 3 |  |
| >High School | 9 | 18 | 27 |  |
| **Variables** | | | | |
| ***Individual*** | | | | |
| **Psychosocial stress (GPSS)** | 4.8 (4.0) n=11 | 7.5 (5.5)  n=19 | 6.5 (5.1) n=30 | 0.17 |
| **Anxiety symptoms** | 10.7 (4.2) n=12 | 11.3 (6.4)  n=17 | 10.9 (5.6) n=28 | 0.70 |
| **Depressive symptoms** | 15.8 (4.6)  n=11 | 15.0 (4.4)  n=20 | 15.0 (4.1) n=30 | 0.63 |
| **Sleep Quality (PSQI)** | 5.9 (3.3) n=9 | 6.6 (5.1)  n=16 | 6.2 (4.4) n=24 | 0.70 |
| **High effort coping: John Henry** | 49.18 (4.8) n=12 | 47.4 (6.7)  n=20 | 48.12 (6.1) n=30 | 0.43 |
| ***Interpersonal*** | | | | |
| **Discrimination** | 19.8 (6.0) n=12 | 23.0 (8.3)  n=20 | 31.7 (7.6) n=31 | 0.26 |
| ***Structural*** | | | | |
| **Area Deprivation (ADI)** | 4.6 (1.8) n=12 | 5.3 (2.8)  n=19 | 5.1 (2.5) n=20 | 0.43 |
| **Distance George Floyd Square**, km | 35.5 (47.8) n=12 | 15.9 (20.6) n=19 | 20.45 (30.6) n=30 | 0.13 |

All values mean (standard deviation, SD) unless otherwise specified.

ADI = Area Deprivation Index; GPSS = Global Perceived Stress Scale; PSQI = Pittsburgh Sleep Quality Index.

**Table S5.** Linear Regression Analyses of Stressful Exposures on Cortisol Dynamic Range (CDR) Outcome (continuous) of Black Men in the FAITH! Heart Health+ Study

| **Independent variable (exposure)** | **Coefficient** | ***P*-value** | **95%CI** |
| --- | --- | --- | --- |
| ***Individual*** | | | |
| **Psychosocial stress (GPSS)** | 0.09 | 0.20 | -0.06, 0.24 |
| **Anxiety symptoms** | 0.08 | 0.21 | -0.05, 0.21 |
| **Depressive symptoms** | 0.04 | 0.53 | -0.10, 0.18 |
| **Sleep Quality (PSQI)** | -0.13 | 0.90 | -0.24, 0.21 |
| **High effort coping:**  **John Henryism** | 0.10 | 0.09 | -0.02, 0.21 |
| ***Interpersonal*** | | | |
| **Everyday discrimination** | 0.04 | 0.42 | -0.06, 0.13 |
| ***Structural*** | | | |
| **Area Deprivation (ADI)** | 0.05 | 0.76 | -0.27, 0.36 |
| **Distance from George Floyd Square**, km | 0.001 | 0.81 | -0.01, 0.13 |

ADI = Area Deprivation Index; GPSS = Global Perceived Stress Scale; PSQI = Pittsburgh Sleep Quality Index.

Note: Adjusting for income did not change the results for Distance from George Floyd Square (B=-0.005, *p*=0.48, 95%CI [-0.02, 0.01]).
